# Alzheimer’s disease mortality among Middle Eastern and North African immigrants in the United States

**DOI:** 10.1002/alz.086204

**Published:** 2025-01-09

**Authors:** Tiffany B Kindratt, Ann O Amuta

**Affiliations:** ^1^ University of Texas at Arlington, Arlington, TX USA; ^2^ Texas Woman’s University, Denton, TX USA

## Abstract

**Background:**

Middle Eastern and North Africa (MENA) adults living in the US experience a higher burden of Alzheimer’s disease (AD) compared to White adults. AD mortality among MENA adults has not been examined. The gap in the literature is because MENA individuals in the US are defined as part of the White race group despite there being differences in health outcomes and lived experiences between MENA and White populations with European origins. Their health also differs from other minoritized immigrant groups, specifically Black African immigrants. The aim of this study is to estimate AD mortality among foreign‐born MENA adults compared to US‐ and foreign‐born White and Black adults.

**Methods:**

Data from publicly available 2000‐2018 National Health Interview Survey and National Death Index Linked Mortality Files through 2019 were analyzed (ages 18+ years). The sample was limited to foreign‐born MENA and US‐ and foreign‐born White and Black adults with a final mortality status captured (n = 763,803). The sample comprised 608,889 US‐born White, 125,803 US‐born Black, 18,751 foreign‐born White from Europe/Russia, 5,172 foreign‐born MENA, and 5,188 foreign‐born Black African adults. All‐cause mortality (%, per 100,000 persons) and AD‐specific underlying cause of death mortality rates (%) were calculated.

**Result:**

All‐cause mortality rate for foreign‐born MENA adults was 5.2%. This was higher than foreign‐born Black (2.3%) but lower than foreign‐born White (13.0%), US‐born Black (12.9%), and US‐born White (13.6%) adults. All‐cause mortality (per 100,000 persons) was highest among US‐born White (13,630) and lowest among foreign‐born MENA (5,240) and Black (2,332) adults. AD was the sixth highest underlying cause of death (4.1%) among MENA immigrants. AD‐specific mortality was similar to foreign‐born White (4.1%) and higher than US‐born White (3.5%) and Black (2.3%) adults.

**Conclusion:**

Findings suggest that foreign‐born MENA adults in the US have a greater burden of AD as an underlying cause of mortality compared to US‐born White and Black adults. In January 2023, the US federal government proposed changes to how race/ethnicity is collected. The new categories include a MENA checkbox to disaggregate this group from White individuals. Our study aligns with the proposed changes to better capture mortality burdens among this minoritized population.

